# Orthorexic eating behaviors are not all pathological: a French validation of the Teruel Orthorexia Scale (TOS)

**DOI:** 10.1186/s40337-023-00764-5

**Published:** 2023-04-28

**Authors:** Clotilde Lasson, Amélie Rousseau, Siobhan Vicente, Nelly Goutaudier, Lucia Romo, María Roncero, Juan Ramón Barrada

**Affiliations:** 1 Laboratoire CERPPS (Centre d’Etudes Et de Recherches en Psychopathologie Et Psychologie de La Santé), Université Toulouse-Jean Jaurès, UT2J, 5 allées Antonio Machado, 31058 Toulouse, France; 2 Centre de Recherches Sur La Cognition Et L’Apprentissage–CeRCA-(CNRS, UMR 7295) MSHS, Université de Poitiers, 5 Rue Théodore Lefebvre, 86073 Poitiers Cedex 9, France; 3 UR4430 CLIPSYD, Université Paris Nanterre; HU Raymond-Poincaré, Inserm CESP 1018 UPS, Nanterre, France; 4 Departamento de Personalidad, Evaluación y Tratamientos Psicológicos, Facultad de Psicología, Universitat de València, Valencia, Spain; 5 Departamento de Psicología y Sociología, Universidad de Zaragoza, Zaragoza, Spain

**Keywords:** Orthorexia, Healthy orthorexia, Orthorexia nervosa, Psychometric evaluation, Healthy eating, Eating disorders, Obsessive–compulsive disorders

## Abstract

As no French validated measurement tool distinguishing healthy orthorexia (HeOr) from orthorexia nervosa (OrNe) currently exists, this study aimed at examining psychometric properties of the French version of the Teruel Orthorexia Scale (TOS). A sample of 799 participants (Mean [SD] age: 28.5 [12.1] years-old) completed the French versions of the TOS, the Düsseldorfer Orthorexia Skala, the Eating Disorder Examination-Questionnaire, and the Obsessive–Compulsive Inventory-Revised. Confirmatory factor analysis and exploratory structural equation modeling (ESEM) were used. Although the bidimensional model, with OrNe and HeOr, of the original 17-item version showed an adequate fit, we suggest excluding items 9 and 15. The bidimensional model for the shortened version provided a satisfactory fit (ESEM model: CFI = .963, TLI = .949, RMSEA = .068). The mean loading was .65 for HeOr and .70 for OrNe. The internal consistency of both dimensions was adequate (α_HeOr_ = .83 and α_OrNe_ = .81). Partial correlations showed that eating disorders and obsessive–compulsive symptomatology measures were positively related to OrNe and unrelated or negatively related to HeOr. The scores from the 15-item French version of the TOS in the current sample appears to present an adequate internal consistency, pattern of associations in line with what was theoretically expected, and promising for differentiating both types of orthorexia in a French population. We discuss why both dimensions of orthorexia should be considered in this area of research.

## Abstract (summary)

Orthorexia ('right appetite', from the Greek) covers two dimensions: (1) orthorexia nervosa (OrNe), a strong preoccupation with healthy diet with negative emotional, cognitive, and/or social consequences while trying to approach this goal and when the eating behavior deviates from it, and (2) healthy orthorexia (HeOr), which can be defined as a healthy interest in diet, (self-assessed) healthy behavior with regard to diet and eating healthily as part of one’s identity. OrNe is not yet indexed into mental disorder classifications. Some prominent measurement tools in the area of orthorexia present important limitations: it is unclear if they validly assess OrNe and they do not tap HeOr by design. To overcome these limitations, a promising self-reported instrument was developed: the Teruel Orthorexia Scale (TOS), a bidimensional questionnaire whose structure has been replicated in different samples. Our research based on 799 participants aimed to adapt this instrument in a French speaking population. The results revealed that the French version of the TOS is an empirically supported tool allowing to differentiate both forms of orthorexia (healthy and nervosa). It also suggests that OrNe is associated with psychopathological symptoms while opposite patterns were found with HeOr. We discuss the importance of measuring both orthorexia dimensions.

## Background

The health benefits of a healthy lifestyle including healthy eating and physical activity have been widely reported over the years (e.g., [61]. However, for some individuals, ideas and interest about healthy eating might become pathological, reaching an obsession with eating they deem healthy, feeling guilty or punishing themselves for deviating from their own standards of healthy diet, and with negative impact in their social lives (orthorexia nervosa, OrNe, [18]. For other individuals, their interest in healthy eating is not associated with psychological or social problems (healthy orthorexia, HeOr) and may even be negatively associated with pathological symptoms [6].

Thus, orthorexia ('right appetite', from the Greek) comprises two dimensions that can and should be differentiated. While HeOr can be defined as a healthy interest in diet, (self-assessed) healthy behaviors with regard to diet, and eating healthily as part of one’s identity [6, 21], the core element of OrNe is a strong preoccupation with healthy diet with negative emotional, cognitive, and/or social consequences while trying to approach this goal and when the eating behavior deviates from it. As Roncero et al. [52] noted: “HeOr is not a measure of healthy eating and, thus, should not be confused with it. People who score high on HeOr indicate that healthy eating is an important part of their life and that they devote time and energy to it. However, their beliefs about healthy eating may not coincide with an objective or real definition of healthy eating. For this reason, the two terms should not be taken as synonyms because a high score on HeOr does not necessarily mean that the person is eating healthily” (p. 2).

Despite that these past few years, several diagnosis criteria proposals were made for OrNe (with converging criteria being: obsessional preoccupation with eating "healthy food", severe distress, impairment of physical health, and impairment of social, academic or vocational functioning) [9, 12, 13, 24, 46], OrNe is not yet indexed into mental disorder classifications and the way to categorize it is currently under debate. Some studies suggested that OrNe belongs to the eating disorder spectrum [7, 16, 55], considering that OrNe is close to anorexia nervosa, bulimia nervosa, and avoidant/restrictive food intake disorder. Others have indicated the overlap with obsessive–compulsive and related disorders or even with anxiety disorders [16, 55]. The convenience of distinguishing OrNe from other eating disorders have even been questioned. Meule and Voderholzer [42] considered that “it is not clear whether ON [OrNe] is actually a diagnostic entity that is distinct from established eating disorders” (p. 1). From our point of view, Meule and Voderholzer [42] confused overlap (and it is clear that OrNe and other eating disorders are related) with equivalence. For instance, Zickgraf and Barrada [64] showed that, controlling for a general measure of eating disorders, orthorexia scores were still related to additional variables. Bhattacharya et al. [11] have argued that OrNe is a "new cultural manifestation" of anorexia nervosa. From our point of view, current knowledge about OrNe allows us to discard this. Indeed, while a key diagnostic criteria of anorexia nervosa in the DSM-5 [1] is a significant low body weight, previous research has shown that OrNe is unrelated to body mass index (e.g., [6, 21]. This lack of consensus on its categorization or even the need for a new diagnosis is notably due, at least in part, to methodological issues in the orthorexia field with important validity problems with some prominent measurement tools [45].

So far, the main focus of the orthorexia literature has been on its pathological form, OrNe [8], for a review see: [41], with the majority of studies relying on the ORTO-15 (or any of its derivatives) to assess the construct [22, 23]. The ORTO questionnaires have been widely criticized [5, 26, 43, 45, 49, 53]. Donini et al. [23] pointed out the lack of efficiency of their questionnaire to distinguish healthy eating behaviors from a pathological obsessive interest in healthy eating. Although a shorter and revised version has been proposed [51], the main problem is still present: questionable content validity. Consider the items "In the last three months, did thoughts of food make you feel guilt, ashamed and anxious?" and "Does thinking about food excessively worry you for more than three hours a day?". "Thinking about food", clearly, is not tapping specifically concerns about healthy eating. Those items could be considered as a general measure of eating disorders, while the ORTO is supposed to measure OrNe. The results of studies based on these assessment tools should then be considered with caution.

To overcome these limitations, several other measurement tools were developed for the assessment of OrNe, mainly, including the Eating Habits Questionnaire (EHQ; [30]) and the Düsseldorfer Orthorexia Skala (DOS, [10]). Both share a characteristic: their internal structure is unclear. It is not clear how many dimensions are being assessed or how to interpret those factors. For the EHQ, solutions of two [33], three [30, 31, 47], and four factors [32] have been proposed. Even for solutions with the same number of factors, neither the distribution of items by factors, nor their theoretical interpretation were equivalent. While Meule et al. [43] found an adequate fit for the original structure on a German sample of 511 participants, no other models were tested. Regarding the DOS, data did not support the presence of a single dimension for the Arabic [33], German [10], English [20], and Chinese [34] versions of the scale. The model fit of the unidimensional solution in the French sample was not satisfactory [37]. In the Spanish version, a unidimensional model offered an excellent fit [48]. For the Chinese version, a three-dimensional solution was considered, with factors labeled Obsession in Healthy Food, Adherence to Nutrition Rules, and Emotional Symptoms. Meule et al. [43] obtained a good fit for the unidimensional solution. Although multidimensional solutions have been commonly proposed for the EHQ and the DOS, it has been a common practice to compute just a single total score. For a recent review showing how these instruments have been used, see Atchison and Zickgraf [3].

Importantly, the DOS and the EHQ could be inadvertently covering both HeOr and OrNe, while they are supposed to be only assessing OrNe [33, 65]. Two specific items from the EHQ: "I am more informed than others about healthy eating" or "Eating the way I do gives me a sense of satisfaction" are currently considered as indicative of OrNe. Nevertheless, why they should be considered as indications of OrNe is difficult to see, but it has been used as such in many publications. The same can be said about these items from the DOS: "Eating healthy food is more important to me than indulgence/enjoying the food" or "I have certain nutrition rules that I adhere to". Considering that the pattern of associations of OrNe and HeOr with third variables is different, combining items from these two dimensions into a single total score can lead to results difficult to interpret.

Recently, Barrada and Roncero [6] proposed to no longer consider orthorexia as a unidimensional construct but as a bidimensional construct with not only a pathological form (OrNe,e.g., “I feel overwhelmed or sad if I eat food that I consider unhealthy”) but also an healthy form (HeOr; e.g., “I think that my way of eating is healthier than that of most people”) and developed a promising self-report questionnaire called the Teruel Orthorexia Scale (TOS). As Atchison and Zickgraf [3] noted, "the Teruel Orthorexia Scale (TOS) is the only one of the currently available self-report instruments designed to measure both healthy orthorexia and orthorexia nervosa, and differentiate between these two constructs". Its structure has been replicated across different samples and languages.

The TOS was originally developed in Spanish [6] showing a clear bidimensional structure and its scores indicated good psychometric properties. Further studies have examined the psychometric properties of the bidimensional model of orthorexia in German [58], Arabic [44], Portuguese [50], and English [19, 64] versions and cross-validated the Spanish version [8]. While some studies have used exploratory techniques [6] [CFI = 0.965, TLI = 0.954, RMSEA = 0.060]; [8] [two samples, CFI = 0.960/0.973, TLI = 0.947/0.965, RMSEA = 0.070/0.062]; [19] [CFI = 0.98, TLI = 0.97, RMSEA = 0.06]; [64] [two samples; CFI = 0.978/0.967, TLI = 0.971/0.957, RMSEA = 0.078/0.057]), others have used confirmatory factor analysis (CFA) [58] [CFI = 0.873, RMSEA = 0.085]; [44] [CFI = 0.954, RMSEA = 0.069]; [50] [CFI = 0.94, TLI = 0.93, RMSEA = 0.09]).

It can be seen that those studies using exploratory techniques have obtained better model fits. CFAs present an important drawback with respect to exploratory analysis to test the internal structure of a questionnaire. In the most common way of using CFAs, secondary loadings are fixed to zero, that is, there are many parameters fixed to a value different from what is plausible in the population, as we can expect secondary loadings to be minor, but not nul. These restrictions imply that the recovered parameters, both loadings and interfactors correlations, are distorted. Although the presence of relevant cross-loadings can be detected via modification indices in CFA, it is common that researchers do not check relevant areas of localized strain if an adequate fit for the overall model is found [15]. Exploratory techniques tend to show a better model fit, but, more importantly, can better recover the underlying structure [2].

The original validation study of the TOS [6] indicated the presence of minor, but not zero, secondary loadings. Overall, the different studies have supported the internal structure of the TOS, although with some nuances. In their English validation, Chace and Kluck [19] suggested removing Item 13 (“I would rather eat a smaller portion of healthy food than get full from food that may not be healthy”), as it showed poor differentiation between the factors, and found that Item 9 (“My concern with healthy eating takes up a lot of my time”) presented a secondary loading over 0.30. Zickgraf and Barrada [64] worked with two samples, both English versions of the TOS. High secondary loadings (over |.30|) were found for Item 1 (“I feel good when I eat healthy food”; sample 1), Item 9 (sample 2), and Item 15 (“I try to convince the people in my life to follow my healthy eating habits”; sample 1), with almost equal loadings for this item in sample 2. In a Lebanese sample, Mhanna et al. [44], with a CFA, found that OrNe and HeOr factors showed a high correlation (*r* = 0.74), much higher than that found with exploratory techniques (e.g., *r* = 0.43 in [6], *r* = 0.44/0.49 in [8]. As we have said, fixing the secondary loadings to zero can distort the recovered interfactor correlations. These results stress that using exploratory approaches is more adequate, as there are reasons to expect secondary loadings clearly different from zero. Regarding exploratory approaches for testing the internal structure of questionnaires, we direct readers to several publications [39, 40, 56, 57].

The studies using the TOS have provided evidence for the validity of the two-dimensionality of orthorexia. Both factors relate differently and oppositely to various psychopathology variables, suggesting that HeOr is not an "attenuated" OrNe factor or a risk factor, but that they are different dimensions. Studies have shown that HeOr was positively correlated with well-being, positive affect, dispositional mindfulness, and negatively correlated with psychopathology (eating disorders, obsessive–compulsive disorder, negative affect, antagonism, disinhibition, psychoticism) and BMI. On the other hand, OrNe was positively correlated with psychological distress, restrained eating, obsessive–compulsive symptoms, perfectionism, low physical self-esteem, negative affect, detachment, disinhibition, psychoticism, weight control, health anxiety, substance use, sedentary behavior, and negatively correlated with dispositional mindfulness [6, 8, 19, 21, 52, 59, 64]. HeOr was also positively correlated with nutrition knowledge and diet quality whereas OrNe was negatively correlated with nutrition knowledge and diet quality [64]. These patterns of associations were clearer when partial correlations were used, that is, when the partial overlap between OrNe and HeOr (Pearson correlation between both dimensions typically in the range 0.30–0.50) was statistically adjusted. In addition, a high stability of both dimensions has been observed over an 18-month interval, with a correlation above 0.70 for both factors [6].

So far, the TOS has not been translated and validated in French language population. The aim of this study was thus to adapt the TOS into French language and examine its psychometric properties (internal consistency, factor structure, convergent and discriminant validities) in French population as to provide an empirically supported tool to start studying orthorexia both in its healthy and pathological forms in French population, without considering all healthy eating concerns as pathological. Participants also provided responses to another measure of orthorexia, the DOS [37]. To assess the convergent and discriminant validities of the translated TOS, participants also completed measures of eating disorders (Eating Disorder Examination-Questionnaire [17]), and obsessive–compulsive disorder (OCD, Obsessive–Compulsive Inventory-Revised, [63]) in their French versions. Our first hypothesis was that the French version of the TOS would replicate the bidimensional structure of the original Spanish TOS as well as equivalent psychometric properties. In line with Zickgraf and Barrada [64], as a second hypothesis, we expected that both TOS subscales would be correlated with the DOS, but with OrNe to a greater extent. Although the internal structure of the DOS is unclear, we considered relevant to include this widely used measure in our study to try to understand what this instrument is measuring, as several studies have used it. As prior findings evidenced that DOS items can be a mixture of OrNe and HeOr, the correlations of TOS scores and DOS scores cannot be interpreted as an evidence of convergent validity. Based on results of previous studies using the TOS, our third hypothesis was that OrNe would be positively linked to eating and obsessive–compulsive symptomatology, whereas HeOr would be negatively associated with these variables, after adjusting for the other orthorexia dimension. Recently Atchison and Zickgraf [3] noted that "[…] the true nature of the relationship between ON [OrNe] and eating disorder symptoms may be best explored in study designs that control for the closely related construct of healthy orthorexia. Because of this, statistically adjusting for healthy orthorexia may be necessary to accurately describe the relationship between ON [OrNe] and disordered eating” (p. 2). The convenience of adjusting for the other orthorexia dimension to more accurately describe the associations with different variables can and should be generalized. By doing so, associations are easier to interpret, as the shared variance between OrNe and HeOr is removed. Different studies that have simultaneously considered OrNe and HeOr have used this approach (e.g., [6, 33, 52, 64]). Partial correlations were preferred over other analytical alternatives as regression models—perhaps more commonly reported—for several reasons. First, Pearson and partial correlations are more easily comparable, as both can range from − 1 to + 1, while this is not the case for regression coefficients (even for standardized coefficients). Second, partial correlations and regression coefficients lead to the same inferential conclusions, as both share identical *p*-values.

## Materials and methods

### Participants and procedure

Participants were recruited via an advertisement posted on Facebook groups of university students and people with an interest in food and nutrition. Potential participants were provided with the link to an online questionnaire and were told that if they agreed to take part in the study, they would be asked to answer questionnaires about their eating habits. Only those who gave their informed consent and were aged 18 or above could access the questionnaire. Participants involved in this study did not receive any compensation. The questionnaires were anonymous. The study protocol was approved by the ethics committee of the University of Lille (Registration number 2021–514-S95).

Participants of this sample were 799 adults (82.9% women, 16.1% men, and 1.0% other genders) aged between 18 and 73 years (*M* = 28.5, *SD* = 12.1). Based on self-reported height and weight, mean BMI was 23.5 (*SD* = 4.96). The majority of participants were students (58.8%).

### Measures

#### Socio-demographic data

Participants provided information about their gender, age, height, weight, and professional status.

#### Teruel Orthorexia Scale (TOS; [6])

This scale assesses orthorexia in two separate dimensions: OrNe (8 items; e.g., “Thoughts about healthy eating do not let me concentrate on other tasks”) and HeOr (9 items; e.g., “My interest in healthy food is an important part of the way I am, of how I understand the world”). Responses are scored on a four-point Likert scale ranging from 0 = *completely disagree* to 3 = *completely agree*.

In the current study, the TOS was translated from Spanish to French by bilingual researchers, using a back-translation procedure [14]. The TOS was first translated into French and then back translated into its original language. The expert committee worked on the discrepancy between the original version and the back translated version to ensure the equivalence of the construct between the original version and the French version as well as the correct understanding of all items. Fifteen freshmen were asked to complete the TOS. Once they responded, a discussion was initiated to ensure that all questions were understandable and that none were problematic. We did not receive any feedback on the meaning and structure of the items.

The final version of the French version of the TOS is presented as an Appendix. Item wording in English can be seen in Table 2.

#### Düsseldorfer orthorexia skala (DOS; [10])

This 10-item scale assesses orthorexic eating behaviors. Responses are scored on a four-point Likert-scale ranging from 1 = *this does not apply to me* to 4 = *this applies to me*. An example item is “If I eat something I consider unhealthy, I feel really bad”. Higher scores are interpreted as indicative of higher levels of orthorexic eating behaviors. We used the French version [37]. Cronbach’s α value was 0.84 in this study. As we have noted in the introduction, the internal structure and interpretation of DOS scores are not completely clear.

#### Eating disorder examination-questionnaire (EDE-Q; [27])

This questionnaire assesses symptoms of eating disorders over the past 4 weeks. Items are scored on two seven-point scales ranging from 0 = *no days* to 6 = *every day* or from 0 = *not at all* to 6 = *markedly*, and are divided into 4 subscales: Restraint (5 items), Eating Concern (5 items), Weight Concern (5 items), and Shape Concern (8 items). Higher scores indicate higher eating or body concerns or behaviors. We used the French version [17]. In this study, Cronbach’s *α* values were 0.83 for Restraint; 0.78 for Eating concern; 0.81 for Weight Concern, and 0.91 for Shape concern.

#### Obsessive–compulsive inventory-revised (OCI-R; [29])

This 18-item self-report scale assesses obsessive–compulsive symptoms. Each item (e.g., “I get upset if others change the way I have arranged things”) is scored on a five-point Likert scale ranging from 0 = *not at all* to 4 = *extremely*. We used the French version [63]. Cronbach’s α was 0.89 in this study.

### Data analysis

The internal structure of the TOS scores was analyzed with a CFA and an Exploratory Structural Equation Model (ESEM) [2]. ESEM is a technique that, unlike CFA, estimates all secondary loadings and, unlike exploratory factor analysis, allows the incorporation of correlated uniquenesses. Previous analyses have found that, although the internal structure of the TOS was clear and theoretically interpretable, secondary loadings cannot be expected to be equal to zero. In cases like this, a CFA would distort the recovered parameters and could achieve inadequate model fit that could lead to the rejection of theoretically useful and interpretable models. If an adequate model fit was reached, fixing to zero parameters whose value is not null in the population would still distort the recovered parameters. We used CFA to show the limitations of this analytical approach in cases like the TOS scores. Previous validation studies of this scale have used CFAs, with the exposed limitations. We consider that one of the reasons for doing so may be that ESEM is not still well-known in this area of research. If we only computed ESEM analysis (although this would be a reasonable psychometric approach), we would miss the opportunity to show the comparison between these two approaches. For comparability, we also tested a unidimensional model for the DOS scores. (For unidimensional models CFA and ESEM are equivalent). We defined a single factor as this has been the standard interpretation of this scale. As we used a four-point Likert scale and responses should be considered as ordinal [28], models were analyzed using robust weighted least squares (WLSMV estimator in M*Plus*). Geomin rotation was used. According to conventional cut-offs [36], comparative fit index (CFI) and Tucker Lewis index (TLI) with values greater than 0.95 and RMSEA less than 0.06 were indicative of a satisfactory fit. It should be noted that these cutoffs were developed for CFAs with continuous responses, so these values should be interpreted with caution [62]. Additionally, these cut-off values should be considered as rough guidelines and not interpreted as “golden rules” [38]. Local fit was evaluated using modification indices. Internal consistencies of the TOS scores for both dimensions were computed with Cronbach's alpha.

We computed descriptive statistics (means, standard deviations, skewness, and kurtosis) and correlations between the two orthorexia dimensions and the six additional variables (DOS score, four EDE-Q scores, and OCI-R score). We present Pearson correlations for all the bivariate associations. We compared the correlation sizes for the six additional variables and the two orthorexia factors with Hittner, May, and Silver's technique [35], a modification of Dunn et Clark’s one [25], that is, we computed if the subtraction of the correlations with OrNe and HeOr were different from zero. Considering that OrNe and HeOr can be expected to be partially overlapped, we computed partial correlations for all the associations between orthorexia and other variables while controlling for the other orthorexia dimension. By doing so, we expected to better assess the associations of HeOr (or OrNe) with the six psychopathological indicators while controlling for OrNe (or HeOr). The analyses were performed with MPlus 8.4 and R 4.1.1.

## Results

### Internal structure and reliability

Results of model fit for the different models can be found in Table 1. As we anticipated, the model fit of the CFA for the TOS scores was clearly inadequate (CFI = 0.881, TLI = 0.863, RMSEA = 0.106). This model was greatly improved when scores were modeled with an ESEM, that is, when cross-loadings were also estimated (CFI = 0.948, TLI = 0.932, RMSEA = 0.075), although fit was still below commonly used cut-off points. As can be seen in Table 2, in this model each item presented its higher loading in the intended factor and all cross-loadings could not be considered as relevant (≤ 0.30), but the one for Item 9 (cross-loading = 0.40). We decided to remove this item for two reasons: first, empirical, given that cross-loading; second, theoretical. While concern with healthy eating is a core element of OrNe, devoting long time is not, as indicated by the fact that Item 2 ("I spend a lot of time buying, planning, and or/preparing food so my diet will be as healthy as possible") clearly loads in HeOr.

**Table 1:** Goodness of fit indices for the different models

Models	$\chi^{2}$	*df*	CFI	TLI	RMSEA
TOS CFA	1181.3	118	.881	.863	.106
TOS ESEM	565.2	103	.948	.932	.075
TOS ESEM No Items 9 & 15	353.9	76	.963	.949	.068
DOS	683.0	35	.877	.842	.152

*df* = degrees of freedom; *CFI* = comparative fit index; *TLI*= Tucker-Lewis index; *RMSEA* = root mean square error of approximation; *TOS* = Teruel Orthorexia Scale; *DOS* = Düsseldorfer Orthorexia Skala; *CFA* = confirmatory factor analysis; *ESEM* = exploratory structural equation model

All *p*-values for the $\chi^{2}$ test were < .001

**Table 2:** Item loadings of the initial and final version of the Teruel Orthorexia Scale-French version

	Initial Version		Final Version	
	HeOr	OrNe	HeOr	OrNe
1. I feel good when I eat healthy food	**0.64**	0.00	**0.65**	0.01
2. I spend a lot of time buying, planning, and or/preparing food so my diet will be as healthy as possible	**0.72**	0.09	**0.71**	0.08
3. I think that my way of eating is healthier than that of most people	**0.84**	− 0.19	**0.83**	− 0.18
4. I feel guilty when I eat food that I do not consider healthy	− 0.01	**0.72**	0.00	**0.73**
5. My social relationships have been negatively affected by my concern about eating healthy food	0.18	**0.56**	0.18	**0.57**
6. My interest in healthy food is an important part of the way I am, of how I understand the world	**0.59**	0.23	**0.57**	0.23
7. I'd rather eat a healthy food that is not very tasty than a good tasting food that isn't healthy	**0.47**	0.27	**0.46**	0.28
8. I mainly eat foods that I consider healthy	**0.95**	− 0.18	**0.96**	− 0.17
9. My concern with healthy eating takes up a lot of my time	**0.40**	**0.51**	–	–
10. I am concerned about the possibility of eating unhealthy foods	0.21	**0.60**	0.20	**0.59**
11. I don't mind spending more money on food if I think it's healthier	**0.54**	0.02	**0.54**	0.03
12. I feel overwhelmed or sad if I eat food that I consider unhealthy	− 0.01	**0.83**	0.00	**0.84**
13. I would rather eat a smaller portion of healthy food than get full from food that may not be healthy	**0.51**	0.25	**0.51**	0.27
14. I avoid eating with people who do not share my ideas about healthy eating	0.28	**0.51**	0.21	**0.52**
15. I try to convince the people in my life to follow my healthy eating habits	**0.46**	0.19	–	–
16. If I ever eat something I consider unhealthy, I punish myself for it	− 0.05	**0.85**	− 0.07	**0.86**
17. Thoughts about healthy eating prevent me from concentrating on other tasks	0.03	**0.82**	0.00	**0.82**

*HeOr* = Healthy Orthorexia; *OrNe* = Orthorexia Nervosa

Loadings in bold indicate unsigned loadings above |.30|

For both versions, *r*_HeOr,OrNe_ = .32

The higher modification index (MI = 108.4) in this model indicated that model fit would be improved if we added the correlation of item uniquenesses from Item 14 ("I avoid eating with people who do not share my ideas about healthy eating”) and Item 15. These two items belong to different factors, and we could not find any theoretical justification –apart from the general reference to 'people'– for the incorporation of this new parameter. Considering that Item 15 has been found to be problematic in previous analysis of the TOS [64], we decided to remove that item. By doing so, we avoided incorporating correlated uniquenesses without any a priori expectation and theoretical justification for doing so.

For the shortened TOS, without Item 9 and Item 15, model fit was improved, although with TLI and RMSEA results slightly over the cut-point (CFI = 0.963, TLI = 0.949, RMSEA = 0.068). As no modification index clearly stood out (maximum MI = 25.3), we considered that no respecification of the model was to be considered. The loadings in this model basically followed what was expected. For HeOr, the mean loading was 0.65 (maximum = 0.96, "I mainly eat foods that I consider healthy"; minimum = 0.46, "I'd rather eat a healthy food that is not very tasty than a good tasting food that isn't healthy"). For OrNe, the mean loading was 0.70 (maximum = 0.86, "If, at some point, I eat something that I consider unhealthy, I punish myself for it"; minimum = 0.52, “I avoid eating with people who do not share my ideas about healthy eating”). The mean unsigned cross-loading was small, 0.13, with a maximum 0.28 for Item 7 (“I'd rather eat a healthy food that is not very tasty than a good tasting food that isn't healthy”).

For both the initial TOS and final versions the latent correlation between HeOr and OrNe was the same, 0.32. Importantly, the correlation between the initial and final versions was 0.99 for both HeOr and OrNe. The internal consistency of both TOS dimensions was adequate (initial version: α_HeOr_ = 0.83 and α_OrNe_ = 0.83; shortened version: α_HeOr_ = 0.83 and α_OrNe_ = 0.81).

In accordance with previous results, the fit of the unidimensional model of the DOS scores was clearly inadequate (CFI = 0.877, TLI = 0.842, RMSEA = 0.152).

### Descriptives and associations with other variables

Table 3 shows the descriptive statistics and the associations among the different variables. As could be expected, the mean of HeOr was higher than for OrNe. Also, HeOr presented a more symmetrical shape than OrNe, with a positive skewness for the latter.

**Table 3:** Descriptive statistics and correlations

	Descriptives				Pearson correlations							Differences in correlations		Partial correlations	
	*M*	*SD*	*Sk*	*K*	1	2	3	4	5	6	7	*diff r*	*p diff*	HeOr	OrNe
1. TOS HeOr	12.84	4.69	− 0.26	− 0.22								–	–	–	–
2. TOS OrNe	5.23	3.78	0.88	0.55	**.40**							–	–	–	–
3. DOS	18.30	5.29	0.72	0.50	**.57**	**.73**						**.16**	** < .001**	**.44**	**.66**
4. EDE-Q Restraint	7.18	7.80	1.07	0.23	**.24**	**.48**	**.56**					**.25**	** < .001**	.05	**.44**
5. EDE-Q Eating Concern	4.37	5.90	1.66	2.14	.02	**.52**	**.44**	**.54**				**.50**	** < .001**	− **.24**	**.56**
6. EDE-Q Shape Concern	20.80	14.20	0.19	− 1.19	.02	**.43**	**.40**	**.62**	**.67**			**.41**	** < .001**	− **.19**	**.46**
7. EDE-Q Weight Concern	11.24	8.44	0.37	− 1.00	.02	**.41**	**.39**	**.60**	**.69**	**.91**		**.39**	** < .001**	− **.17**	**.44**
8. OCI-R	38.35	12.59	0.55	− 0.17	− .05	**.31**	**.24**	**.19**	**.39**	**.29**	**.27**	**.36**	** < .001**	− **.20**	**.36**

*HeOr* = healthy orthorexia, *OrNe* = orthorexia nervosa, *diff r* corresponds to the difference *r*(variable,OrNe) – *r*(variable,dimension,HeOr), *p diff* corresponds to the statistical significance of that difference

Partial correlations are controlling for the other orthorexia dimension

Bold values correspond to statistically significant associations and differences, *p* < .05

Both TOS dimensions presented a positive correlation with DOS score, with TOS OrNe showing a higher association (*r* = 0.57 with TOS HeOr; *r* = 0.73 with TOS OrNe). While HeOr presented small correlations with the different EDE-Q and OCI-R scores (*M*_|r|_= 0.07, minimum = 0.02, maximum = 0.24; none statistically significant, but the latter), the associations with OrNe were much higher (*M*_|r|_= 0.43, minimum = 0.31, maximum = 0.52; all statistically significant).

Associations between DOS, EDE-Q and OCI-R with TOS showed significant differences when comparing the coefficients found for HeOr and OrNe [*r*(variable,OrNe) – *r*(variable,HeOr)], that is, all the measures indicative of disorders were more related with OrNe than with HeOr. The size of these differences ranged from 0.16 for DOS to 0.50 for Eating Concern, with all *p*s < 0.001.

When controlling for the other orthorexia dimension, the pattern of partial correlations for HeOr presented interesting differences with respect to Pearson correlations (see Table 3). While the association with DOS scores was reduced from 0.57 to 0.44, the partial correlation still indicated a relevant overlap. For EDE-Q and OCI-R scores, the only positive and statistically significant Pearson correlation was now a near-zero and non-significant partial correlation (from 0.24 to 0.05) and all the other near-zero Pearson correlations showed then negative and statistically significant partial association in the range [–0.24, –0.17]. With respect to OrNe, the association with DOS scores was slightly reduced when controlling for HeOr (from 0.73 to 0.66). The partial correlations with EDE-Q and OCI-R scores increased for all variables except for Restraint.

## Discussion

To date, several orthorexia measurement tools have been translated and validated in French language (i.e., the ORTO-15, [4], the EHQ, [31],and the DOS, [37]). However, all those instruments, arguably, present several limitations [5, 10, 20, 26, 30, 34, 37, 45, 47, 49, 53]. There is evidence that the DOS and the EHQ, simultaneously, tap both OrNe and HeOr [33, 64], but it is still common practice in the area of orthorexia to compute total scores for those scales (see [3] for a review about the associations of those scores with other eating disorders).

In comparison, the TOS presents several advantages. First, it explicitly covers the healthy side of orthorexia. Second, the validity of its scores seems, up to now, clearer. The aim of the current study was thus to examine psychometric properties of the French version of the TOS. To the best of our knowledge, this is the first study proposing a French validation of a questionnaire taking into account both the healthy and pathological forms of orthorexia.

One would expect OrNe and HeOr to represent a continuum, ranging from no interest in healthy eating to an important negative impact of deviance from own healthy eating standards, with an optimal interest as a middle point. However, as noted in previous studies (e.g., [6]), these two factors can and should be separated. In this study, for both the initial TOS and final versions the latent correlation between HeOr and OrNe was the same, that is 0.32. Comparing the correlations showed with both dimensions, they are very different and opposite for most factors, mainly when controlling for the other orthorexia dimension [21, 52, 59, 64]. A person can score high on HeOr without necessarily experiencing distress or interference in their life. This is very important, as it indicates that interest for healthy eating and considering that life-style option as part of own identity is not associated to psychopathology. In fact, HeOr negatively correlates with psychopathology and positively correlates with positive affect [6, 8]. This pattern of associations can be seen in the results found in the present study with the French version of the TOS.

Unlike prior validation studies [44, 50, 59] exploring the bidimensional structure of the TOS using CFA, we used an ESEM allowing estimation of secondary loadings and correlated uniquenesses. As anticipated, the model fit of the CFA for the TOS scores was clearly inadequate and it was greatly improved when using ESEM. Our general advice would be to avoid CFA when cross-loadings can be expected (and this is more the norm than the exception), so exploratory approaches should be favored. Similarly, the CFA conducted on the DOS scores showed an unsatisfactory fit, questioning the unidimensional structure of the measure [37]. Based on ESEM, we obtained a 15-item two-factor solution that provided a good fit with the data and clearly replicated the bidimensional structure found in several TOS validation studies [6, 19, 44, 50, 59, 64]. Nevertheless, while the Portuguese [50], Arabic [44], and one of the English validation studies [64] replicated a 17-item version of the TOS, which was consistent with the original Spanish version [6], we chose to retain a 15-item solution and removed Item 9 and Item 15. Regarding Item 9, cross-loadings were already found in other validation studies [19, 64]. On a theoretical standpoint, we may consider that while “concern with healthy eating” is a core component of OrNe, it can also cover several activities such as planning, shopping or cooking already assessed by Item 2, which is clearly on the HeOr factor. Another important point to emphasize is that this time-consuming component might be considered subjectively depending on people and Item 9 does not specify an amount of time above which a pathological behavior could be considered. Thus, the cross-loading of this item could be explained by its non-specific and vague nature. Regarding Item 15, an English validation study [64] has already questioned its relevance given that reverse patterns of loadings were observed between the two samples recruited in the study. Thus, trying to convince others to consume healthy food would not be specific to one type of orthorexia, suggesting the need to focus on motivations underlying eating habits that could play a part in the development of each type of orthorexia.

Regarding scale properties, in line with previous studies [6, 19, 44, 50, 59, 64], the OrNe and HeOr subscales showed only moderate latent intercorrelations (*r* = 0.32). The internal consistency of both TOS dimensions was adequate (initial version: α_HeOr_ = 0.83 and α_OrNe_ = 0.83; shortened version: α_HeOr_ = 0.83 and α_OrNe_ = 0.81) and of comparable magnitude of values reported in the original Spanish version [6] as well as in other TOS validation studies [19, 44, 59], suggesting that this version of the TOS is an empirically supported tool to explore orthorexia both in its healthy and pathological forms in French samples.

Although both TOS subscales were found to be associated with another measure of Orthorexia Nervosa –the DOS–, providing support for convergent validity, the strongest correlation was observed with the OrNe subscale (*r*_DOS-OrNe_ = 0.73 vs. *r*_DOS-HeOr_ = 0.57). As was noted in previous research [64], if the DOS was a pure marker of OrNe, that is, it was assessing other constructs to a large degree, controlling for TOS-OrNe, TOS-HeOr would be minimally correlated with the DOS. We did not find such associations. This result, combined with the unidimensional model fit with these data and previously reported data, indicates that the DOS is a measure which should be further improved.

Regarding the relationships with measures of disordered eating behaviors and obsessive–compulsive symptoms, as predicted, HeOr and OrNe showed opposite patterns of correlations, which is in line with previous validation studies [6, 64]. As expected, the correlation between HeOr and obsessive–compulsive symptoms (OCI-R) was near-to-zero while it was significant but moderate with OrNe. Interestingly, when controlling for the other dimension, the OCI-R-HeOr partial correlation became significant but negative, however small, with the reverse pattern for the OCI-R-OrNe that slightly increased. These results suggest that OrNe and OCD share common clinical manifestations. According to the literature, the overlapping symptoms might be obsessions, preoccupations specifically oriented towards healthy eating, a considerable amount of time devoted to obsessions and preoccupations (planning, shopping, preparation), and an important rigidity concerning certain rules leading to guilt if not followed.

Concerning the relationships between HeOr and eating disorder symptoms (EDE-Q four subscales: Restraint, Eating Concern, Weight Concern and Shape Concern), the correlations were non-significant except for one –Restraint– which was small, whereas the correlations between EDE-Q subscales and OrNe were all much higher and statistically significant. More interestingly, the partial correlations between HeOr and EDE-Q subscales became all significant and negative, yet small, when controlling for OrNe, except with the Restraint subscale that became a near-to-zero and non-significant partial correlation. On the contrary, the partial correlations between EDE-Q subscales and OrNe all increased, except for the Restraint subscale that slightly decreased. These results confirm previous findings and the two-dimensional conception of orthorexia [6, 19, 44, 64]. One important finding is indeed the negative relationships found here between the HeOr dimension and the eating, shape and weight concern symptoms that are core features of eating disorders. Overall, these findings suggest the importance of distinguishing HeOr from OrNe.

As Valente et al. [60] have pointed, the proposal of OrNe leads to the emergence of a discursive tension: are we pathologizing healthy eating? From our point of view, the researchers in the area of OrNe should acknowledge that we, to some degree, have been “anti-health” and pathologizing healthy eating and healthy life choices. For many years, the main instrument to measure OrNe has been the ORTO-15. Among its items we can find "Are you willing to spend more money to have healthier food?" or "Do you think that on the market there is also unhealthy food?". That is, an economic compromise with healthy food has been used as a marker of a (new proposed) eating disorder. Willing to spend money on health was indicative of pathology. Also, having basic nutritional knowledge (not all what is sold in a market is healthy) has also been used as a marker of eating disorder. As we have already noted, other questionnaires present similar problems. If part of the general public has understood that the proponents of OrNe were trying to pathologize healthy eating [54], we should accept that, to some degree, it has been a fault from our research community.

Clearly separating between what is pathological and what is not, between OrNe and HeOr can clarify both constructs. People highly engaged with healthy eating and no pathological approach to healthy eating (high HeOr, low OrNe) will not recognize themselves with the OrNe items of the TOS. We are offering a label for those that could identify with HeOr (healthy interest in diet, self-assessed healthy behavior with regard to diet, and eating healthily as part of one’s identity).

From our point of view, the bidimensional structure of orthorexia has been useful to advance the understanding of the topic. First, it has offered a theoretical model that has helped to understand the structure of some commonly used questionnaires in the area. Second, it has clearly shown that some elements that, at a superficial inspection, could be considered as elements of OrNe, in fact, are not, like devoting a long time to prepare food or considering that our own diet is healthier than that of most people. This helps us to refine how we conceptualize OrNe. A high engagement with healthy eating is not a problem. The disorder is based on feeling guilty, punishing for eating what is considered unhealthy, or social isolation.

Despite several strengths in the current study (sample size, wide age range, data gathered in the general population, accurate statistical analyses, validated tools for exploring convergent and discriminant validities), some limitations need to be acknowledged. Although the study was conducted on a large sample of participants, there is, as for prior validation studies [19, 44], an over representation of women (82.9%) which limits the representability of the results. Moreover, professional domains of activities and socioeconomic status were not specifically requested, and such lifestyle-related variables may have a considerable influence on nutrition knowledge and food quality availability. In addition, it is noteworthy that data were collected during the COVID-19 pandemic which might have influenced eating habits and/or general mental health. Finally, the cross-sectional design of the study precludes drawing conclusions about the causality between OrNe and syptoms of eating disorders and obsessive–compulsive disorder.

## Conclusions

The current study provides additional evidence for a bidimensional conception of orthorexia and suggests that the TOS, in a 15-item version, could be an empirically supported instrument for differentiating both types of orthorexia (i.e., OrNe vs. HeOr) in a French population. To date, the available measurement tools in French language showed some discrepancies and limited psychometric properties [4, 37]. Beyond such inconsistencies, none of the existing French assessment tools have yet considered a bidimensional approach of orthorexia. Thus, the current validation of the TOS could have important implications for future studies in French speaking populations and could allow the replication of international studies. Regarding clinical implications, our findings suggest the need to provide appropriate psychoeducative information to individuals demonstrating healthy food preoccupation to avoid the development of pathological manifestations. Future studies should include measures of quality of life, distress, motivations, and emotion regulation in order to better characterize the pathological and non-pathological dimensions of orthorexia.

## Data Availability

The data that support the findings of this study are available from the corresponding author (CL), upon reasonable request.

## Appendix-Teruel Orthorexia Scale

Les questions suivantes concernent les idées et les attitudes que vous avez vis-à-vis de l’alimentation. Plus précisément, nous souhaiterions savoir dans quelle mesure il est important pour vous de suivre une alimentation saine ou de consommer des aliments, par exemple, sans gras, sans sel, sans conservateurs, sans additifs ou toute substance que vous considérez comme nocive ou toxique comme les pesticides.

**Table:**

	Pas du tout d’accord	Plutôt pas d’accord	Plutôt d’accord	Tout à fait d’accord
1. Je me sens bien quand je mange des aliments sains				
2. Je passe beaucoup de temps à acheter, à planifier et/ou à préparer la nourriture pour que mon alimentation soit la plus saine possible				
3. Je considère que ma façon de manger est plus saine que celle de la plupart des gens				
4. Je me sens coupable lorsque je mange de la nourriture que je considère comme mauvaise pour la santé				
5. Ma préoccupation à manger sainement a eu un impact négatif sur mes relations sociales				
6. Mon intérêt pour une alimentation saine définit ma manière d’être, de comprendre le monde				
7. Je préfère manger une nourriture saine et peu savoureuse qu’une nourriture savoureuse mais mauvaise pour la santé				
8. Je consomme principalement des aliments que je considère sains				
9. Je suis préoccupé(e) par l’éventualité de manger des aliments mauvais pour la santé				
10. Cela ne me dérange pas de dépenser plus d’argent pour un aliment si je le considère plus sain				
11. Je me sens bouleversé(e) ou triste si je mange des aliments que je considère mauvais pour la santé				
12. Je préfère manger peu, mais de la nourriture saine, que d’être rassasié(e) avec de la nourriture mauvaise pour la santé				
13. J’évite de manger avec des personnes qui ne partagent pas mes idées sur une alimentation saine				
14. S’il m’arrive de manger quelque chose que je considère comme mauvais pour la santé, je me punis pour cela				
15. Des pensées concernant une alimentation saine m’empêchent de me concentrer sur d’autres tâches				

### Calcul des scores

Échelle de réponse: Pas du tout d’accord = 0, pas d’accord = 1, plutôt d’accord = 2, tout à fait d’accord = 3.

TOS—Orthorexie saine = TOS1 + TOS2 + TOS3 + TOS6 + TOS7 + TOS8 + TOS10 + TOS12.

TOS—Orthorexie nerveuse = TOS4 + TOS5 + TOS9 + TOS11 + TOS13 + TOS14 + TOS15.
